# Editorial: Surgical treatment of peripheral neuropathic pain, peripheral nerve tumors, and peripheral nerve injury

**DOI:** 10.3389/fneur.2023.1266638

**Published:** 2023-08-15

**Authors:** Yanzhao Dong, Hui Lu

**Affiliations:** Department of Orthopedics, The First Affiliated Hospital, Zhejiang University, Hangzhou, Zhejiang, China

**Keywords:** peripheral nerve injuries, nerve regeneration, neurotrophic factor, multidisciplinary collaboration, neuropathic pain

Peripheral nerve injuries (PNIs) and peripheral nerve tumors can be devastating, leading to impaired motor and sensory function, causing neuropathic pain, and impacting the overall quality of life for affected individuals. As we face an increasing number of PNI cases, there is an urgent need to focus on innovative approaches to promote nerve regeneration and functional recovery.

In this editorial, we explore the latest breakthroughs in the field of peripheral nerve injury regeneration. From tissue engineering and biomaterials to neural tissue transplantation and nerve guidance conduits, researchers are exploring diverse avenues to bridge the gap and stimulate nerve regrowth (Zhang P. et al.; Yang et al.; Fu et al.).

One of the key themes of this editorial is the role of neurotrophic factors and growth-promoting molecules in stimulating nerve regeneration from PNIs and peripheral nerve tumors. Understanding the intricate signaling pathways involved in nerve repair and harnessing the power of stem cells hold promise in revolutionizing PNI treatment (Li et al.).

Moreover, we delve into the importance of precise and personalized therapeutic strategies for peripheral nerve injury regeneration and neuropathic pain. Tailoring treatments to individual patients' needs can significantly enhance the success rates and optimize functional recovery outcomes.

The editorial also highlights the significance of multidisciplinary collaboration in advancing PNI and peripheral nerve tumor research. Neurologists, neurosurgeons, bioengineers, and molecular biologists must unite to share knowledge and insights, fostering a synergistic approach to tackle the complex challenges of nerve regeneration (Zhou et al.).

We shed light on the role of advanced imaging techniques in assessing nerve regeneration progress, providing valuable insights for clinicians and researchers alike. Additionally, rehabilitation protocols play a vital role in maximizing functional recovery post-injury, and we underscore the importance of comprehensive rehabilitation in the editorial (Fan et al.).

While there have been remarkable strides in the field of peripheral nerve regeneration post PNIs or peripheral nerve tumor, we recognize that there are still hurdles to overcome. Ethical considerations, safety, and long-term efficacy remain paramount in the development of novel therapies.

In conclusion, this editorial calls for continued dedication and investment in peripheral nerve regeneration research. Embracing a holistic and interdisciplinary approach, we can forge a brighter future for individuals facing PNI and peripheral nerve tumor, enabling them to regain function and independence.

## Author contributions

YD: Conceptualization, Investigation, Methodology, Software, Writing—original draft. HL: Conceptualization, Data curation, Formal analysis, Funding acquisition, Methodology, Resources, Supervision, Validation, Writing—review and editing.

